# Synthesis and *in vitro* Cytotoxicity of Novel Ursolic Acid Derivatives

**DOI:** 10.3390/molecules15064033

**Published:** 2010-06-04

**Authors:** Yanqiu Meng, Yanling Song, Zhaokai Yan, Yan Xia

**Affiliations:** Department of Pharmaceutical Engineering, Shenyang University of Chemical Technology, liaoning 110042, China; E-Mails: yanzhaokai110926@163.com (Z.Y.); xiayan1@126.com (Y.X.)

**Keywords:** ursolic acid derivatives, structure modification, anti-tumor activity

## Abstract

In an effort to improve potential hepatoprotective and anti-tumor activities, eight novel ursolic acid (UA) derivatives were designed and synthesized with substitution at positions of C-3, C-11and C-28 of UA. Their structures were confirmed using IR, MS and ^1^H-NMR and elemental analysis. Their *in vitro* cytotoxicity against various cancer cell lines (HeLa, SKOV3 and BGC-823) was evaluated by the standard MTT assay. Among them, compound 13 exhibited more potent cytotoxicity than ursolic acid.

## 1. Introduction

Ursolic acid (**1**, UA) is a pentacyclic triterpene compound isolated from many types of medicinal plants [1]. It has been reported to possess a wide range of pharmacological properties, including antiinflammatory, antiallergic, antibacterial, antiviral and antitumor activities. Among these interesting biological activities, the most intriguing property is the high cytotoxic activity. In 1990, it has been ranked as one of the most promising tumor preventive medications by Japanese authors [2].

UA and their derivatives have been reported to inhibit interferon-induced NO production capacity, and the activities would be significantly enhanced by introduced methoxycarbonyl, carbonyl, or cyano-functional groups [3,4,5,6,7]. Based on the reports that the ester functionality at C-3 is essential for the pharmacological activities of pentacyclic triterpenes [8], and a hydrogen donor group at either C-3 position and/or C-28 position of ursolic acid is essential for the cytotoxic activity [9], a series of UA derivatives have been synthesized and their cytotoxic activities have been evaluated *in vitro* against three cancer cell lines (HeLa, SKOV3 and BGC-823). The results showed that acetylation of the C-3 alcohol together with coupling of a substitued amino group at C-28 results in derivatives having stronger cell growth inhibitory than ursolic acid. In addition, introduction of a carbonyl group on C-11 of UA can also inhibit tumor cell growth.

## 2. Results and Discussion

### 2.1. Synthesis of UA derivatives

**Scheme 1:**
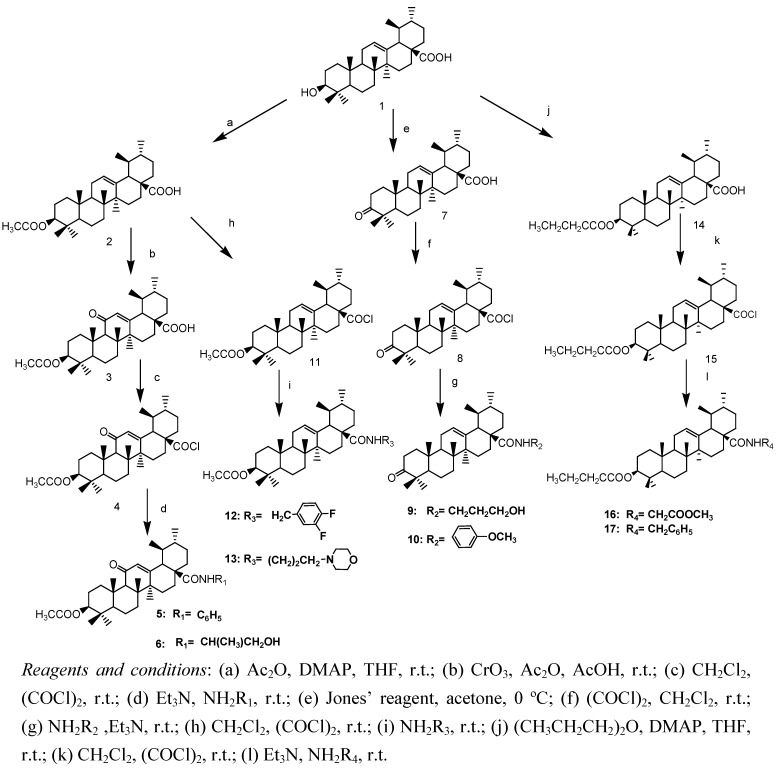
Synthesis of Ursolic acid derivatives.

Ursolic acid was used as the lead compound and the structure modification was done at the positions C-3, C-11 and C-28. The synthetic pathways are shown in Scheme 1.

Compound **2** was prepared by reaction of UA with acetic anhydride in THF in the presence of DMAP, then reacted with chromium trioxide in acetic acid solution containing 5% acetic anhydride to obtain compound **3**, which was treated with oxalyl chloride to yield compound **4**. The crude intermediate **4** was condensed with appropriate amino compounds in the presence of triethylamine to give compounds **5**, **6**. The oxidation of the hydroxyl group of UA afforded the target product **2** in 91% yield. The 3-oxo-ursolic acid **7** was treated with oxalyl chloride to give the 3-oxoursolyl chloride **8**. This intermediate was then condensed with the appropriate amino compound in the presence of triethylamine to give compounds **9**, **10**. Compound **2** was treated with oxalyl chloride to give the 3-O-acetylursolyl chloride **11**. This intermediate was then condensed with the appropriate amino compound in the presence of triethylamine to give the compounds **12**, **13**. Compound **14** was prepared by UA reacted with butyric anhydride in the presence of DMAP in THF. Compounds **16**, **17** were obtained from **14** according to the same method used for synthesizing compound **12**. The target compounds were purified on a silica gel column with petroleum ether/ethyl acetate (or acetone) as eluents and their structures confirmed by mp, IR, MS, ^1^H-NMR and elemental analysis.

### 2.2. Bioactivity

Antitumor activities of compounds **5**, **6**, **9**, **10**, **12**, **13**, **16**, **17** were evaluated *in vitro* using the MTT method against HeLa, BGC-823 and SKOV3 cell lines with UA as the positive control. Tumor cells (200 μL per well) were cultured in 96-well culture plates in RPMI 1640 containing 10% FCS under 5% CO_2_ and 37 ºC. After 24 h, the appropriate test compound was added with different indicated concentrations of 10^-5^, 10^-6^, 10^-7^, and 10^-8^ mol/L, respectively, for another 72 h incubation. Then MTT was added, and absorbance was measured on an ELISA reader at a wavelength of 490 nm. Each test was performed three times. The concentration of compounds which gives 50% growth inhibition corresponds to the IC_50_. The results are summarized in Table 1.

**Table 1:** Inhibitory activity of ursolic acid and its analogues on different cancer cell proliferation.

Comp.	Inhibition rate for different cell (%)^a^			IC_50_(μmol·L^-1^)^b^		
	HeLa	SKOV3	BGC-823	HeLa	SKOV3	BGC-823
1	12.36	9.06	10.20	>10	>10	>10
5	32.39	1.93	31.59	>10	>10	>10
6	29.69	22.57	5.04	>10	>10	>10
9	39.98	8.01	34.84	>10	>10	>10
10	0.15	0.08	0.18	>10	>10	>10
12	17.82	NT	12.89	>10	>10	>10
13	92.97	62.28	85.03	2.71	7.40	4.46
16	41.87	NT	65.12	>10	NT	5.63
17	5.15	4.66	13.17	>10	>10	>10

^a^ Inhibitory percentage of cells treated with 10 μmol/L of each compound for 96 h; ^b^ The agent concentration (uM) that inhibited HeLa cells growth by 50%. NT: not test.

As shown, most of the evaluated compounds esterified at the 3-OH and coupled at C-28 with selected amino acid methyl esters displayed stronger inhibitory activity than UA against the three cancer cell lines. Among them, compound **13** (inhibition rates for HeLa, SKOV3, BGC-823 cells (%), 92.97, 62.28, 85.03, IC_50_ values of 13, 2.71 μM, 7.40 μM, 4.46 μM) displayed the most potent antitumor activity. Compounds **5**, **6**, **9** (inhibition rates (%), 32.39, 29.69, 39.98) presented strong inhibition in the HeLa cell line model. Compound **6** (inhibition rate for SKOV3 cell (%), 22.57) presented strong inhibition in this cell line model. Compounds **5**, **9** (inhibition rates for BGC-823 cell (%) 31.59, 34.84) presented strong inhibition in the BGC-823 cell line model. By comparison of compounds acetylated or butylated at C-3, we can conclude that acetylated compounds exhibit more activity than the butylated ones. Some compounds such as the 3-carbonyl group or 11-carbonyl group UA derivatives might also be important for improving the tumor cell growth inhibitory activity 

## 3. Experimental

### 3.1. General

UA with over 98% purity was purchased from China Chengdu Scholar Bio-Tech.Co., Ltd. Other reagents (analytical grade) were bought from commercial suppliers and used without further puriﬁcation unless otherwise noted. The melting points were determined on an electrically heated X-4 digital visual melting point apparatus and are uncorrected. IR spectra were recorded on a ThermoNicolet 470FT spectrometer. ^1^H-NMR spectra were measured on a Bruker ARX-300 MHz spectrometer at room temperature, with TMS as the internal standard. ESI-MS were determined with Thermo-Finnigan LCQ equipment. Elemental analyses were performed on a Carlo-Erba 1106 Elemental Analysis instrument.

### 3.2. General procedure for the synthesis of N-[3β-acetoxyurs-11-oxo-12-en -28-acyl]-amine compounds ***5**,**6***

A solution of UA (300 mg, 0.658 mmol) in THF (10 mL), pyridine (1 mL), acetic anhydride (1 mL) and a small amount of DMAP was stirred for 4 h at room temperature. When the reaction was complete, the solvent was cncentrated in vacuo and the solids were dispersed in water, then acidified to pH 3–4 with HCl, filtered, washed with water to neutrality, and dried at room temperature to give white solid compound **2**. A mixture of compound **2** (50 mg, 0.1 mmol) with acetic anhydride (0.75 mL), acetic acid (14.25 mL) and CrO_3_ (99.6 mg, 0.1 mmol) was stirred for 4–6 h at room temperature. Water (20 mL) and CH_2_Cl_2_ (20 mL) were added. When stratified, the water layer was extracted twice with CH_2_Cl_2_, combined organic phase, washed with saturated NaHCO_3_, then washed with water to neutrality. Dried over Na_2_SO_4_, filtered, and concentrated in vacuo. Compound **3** was obtained as a light green oil, which was dissolved in CH_2_Cl_2_ (10 mL), oxalyl chloride (0.2 mmol) was added and the mixture stirred 20 h to give compound **4**. Reaction solvent and unreacted oxalyl chloride were eliminated by vacuum. Then the residue was dissolved in cyclonexane (5 mL). This was done twice. Acyl chloride was mixed with CH_2_Cl_2_ (5 mL) and Et_3_N to adjust the pH to 9–10. The solution was stirred for 5 min and amine (0.3 mmol) was added in at room temperature. The reaction completion was detected by TLC. The dichloromethane was eliminated by vacuum, brine (5 mL) was added and the mixture was acidified with concentrated HCl to pH 3–4. A white solid was precipitated, filtered, the filter cake was washed to neutrality with water and dried to give a white solid.

#### 3.2.1. N-[3β-Acetoxy-urs-11-oxo-12-en-28-acyl]aniline *(**5**)*

Compound **4** was reacted with aniline (0.3 mmol) using the general procedure to give compound **5** (27.5 mg, yield: 43%); m.p. 215–217 °C; IR (KBr): 3389, 2930, 1712, 1658, 1610, 1599, 1534, 1499, 1439, 1373, 1260, 754, 691 cm^-1^; ^1^H-NMR (CDCl_3_): 7.10–7.45 (m, 5H, Ar-H), 5.75 (s, 1H, NH), 5.65 (s, 1H, H-12), 4.50 (m, 1H, H-3), 2.06 (s, 3H, CH_3_CO), 0.86, 0.90, 0.95, 0.97, 1.03 (s, each 3H), 0.81 (d, 3H, CH_3_), 0.87 (d, 3H, CH_3_); MS m/z: 605.0 [M+18]^+^; Elemental analysis (%, found) C, 77.68 (77.64); H,8.98 (9.09); N, 2.29 (2.38).

#### 3.2.2. N-[3-Acetoxy-urs-11-oxo-12-en-28-acyl]-4-methylpiperazine *(**6**)*

Compound **4** was reacted 2-amino-1-propanol (0.3 mmol) using the general procedure to afford compound **6** (28mg, yield: 50.3%); m.p. 203–204 °C; IR (KBr): 3441, 2929, 1732, 1657, 1630, 1457, 1366, 1246 cm^-1^; ^1^H-NMR (CDCl_3_): 5.636 (s, 1H, H-12), 5.845–5.861 (d, 1H, NH), 4.537 (m, 1H, H-3), 3.502–3.615 (m, 2H, CH_2_OH), 3.982 (m, H, NCH), 2.063 (s, 3H, CH_3_CO), 1.102 (s, 3H, CH_3_), 1.089 (s, 3H, CH_3_), 0.953 (s, 3H, CH_3_), 0.938 (s, 3H, CH_3_), 0.900 (d, 3H, CH_3_) ,0.882 (s, 3H, CH_3_), 0.798–0.820 (d, 3H, CH_3_), 0.813 (d, 3H, CH_3_); MS *m/z*: 570.5 [M+1]^+^; Elemental analysis (%, found) C,73.71 (73.77); H, 9.73 (9.61); N, 2.46 (2.52).

### 3.3. General procedure for the synthesis of N-[3-oxo-urs-12-en-28-oyl]-amine compounds ***9**,**10***

To a solution of compound **1** (100 mg, 0.22 mmol) in acetone (1.5 mL) Jones’ reagent (0.4 mL) was added dropwise in an ice-salt bath. The reaction mixture was allowed to warm up to room temperature and stirred for 1 hour. After cooling to 0 ºC, 2-propanol (5 mL) was added and the solution stirred at room temperature for 30 minutes. The green precipitate was collected and washed well with acetone. The acetone solution from the combined filtrates were concentrated and dried. By purification on a silica gel column compound **7** was obtained as a white solid. A mixture of compound **7** (50 mg, 0.11 mmol) and oxalyl chloride (0.04 mL) in CH_2_Cl_2_ (2 mL) was stirred at room temperature for 20 h. The mixture was concentrated to dryness under reduced pressure. Cyclohexane (3 × 1 mL) was added to the residue, then concentrated to dryness to yield crude 3-oxoursolyl chloride **8**. To a CH_2_Cl_2_ (4 mL) solution of **8** was added an amine compound (0.44 mmol). The reaction mixture was stirred in presence of Et_3_N at room temperature. The resultant residue was partitioned with 3 mL water, then treated with 2N HCl to pH 3, CH_2_Cl_2_ was removed under vacuum to precipitate a white solid which was filtered and the filter cake was washed with water to pH 7, and dried. The crude was purified on a silica gel column with petroleum ether/ethyl acetate to yield a white powder.

#### 3.3.1. N-[3-Oxo-urs-12-en-28-oyl]-3-amino-1-propanol *(**9**)*

Compound **8** was reacted by using general procedure with 3-amino-1-propanol to give compound **9**. The reaction mixture was stirred at room temperature for 5 h. Elution with petroleum ether/ethyl acetate (v/v) = 3:1; Yield: 45.1%; m.p. 112–114 °C; IR (KBr): 3398, 2927, 1704, 1634, 1527, 1456, 1382, 1077cm^-1^; ^1^H-NMR (CDCl_3_): δ6.23 (s, 1H, NH), 5.33(s, 1H, H-12), 3.60(m, 2H, CH_2_OH), 3.59(br, 1H, NHCHa), 3.17(br, 1H, NHCHb), 2.52 (m, 1H, H-2b), 2.42 (m, 1H, H-2a), 1.12 (s, 3H, CH_3_), 1.10(s, 3H, CH_3_), 0.97 (s, 3H, CH_3_), 0.90 (d, 3H, CH_3_), 0.84 (s, 3H, CH_3_); ESI-MS: 512.5 (M+H)^+^; Elemental analysis (%, found) C, 77.38 (77.45); H,10.41 (10.44); N, 2.81 (2.74).

#### 3.3.2. N-[3-Oxo-urs-12-en-28-oyl]-4-methoxyaniline *(**10**)*

Compound **8** was reacted with p-methoxyaniline using the general procedure to give compound **10**. The reaction mixture was stirred at room temperature for 5 h. Eluted with petroleum ether/ethyl acetate (v/v) = 3:1; Yield: 45.1%; m.p. 112–114 °C; IR (KBr): 3420, 2897, 1650, 1600, 1589, 1490, 1298, 977cm^-1^; ^1^H-NMR (CDCl_3_): δ6.23 (s, 1H, NH), 5.33(s, 1H, H-12), 3.60(m, 2H, CH_2_OH), 3.59(br, 1H, NHCHa), 3.17(br, 1H, NHCHb), 2.52 (m, 1H, H-2b), 2.42 (m, 1H, H-2a), 1.12 (s, 3H, CH_3_), 1.10(s, 3H, CH_3_), 0.97 (s, 3H, CH_3_), 0.90 (d, 3H, CH_3_), 0.84 (s, 3H, CH_3_); ESI-MS: 560.5 (M+H)^+^; Elemental analysis (%, found) C, 78.99 (79.10); H, 9.79 (9.87); N, 2.54 (2.49).

### 3.4. General procedure for the synthesis of N-[3β-acetoxyurs-12-en-28-oyl]-amines ***12**,**13***

A mixture of compound **2** (50 mg, 0.11 mmol) and oxalyl chloride (0.04 mL) in CH_2_Cl_2_ (2 mL) was stirred at room temperature for 20 hours. The mixture was concentrated to dryness under reduced pressure. Cyclohexane (3 × 1 mL) was added to the residue, then concentrated to dryness to yield crude 3-*O*-acetylursolyl chloride **11**. To a CH_2_Cl_2_ (4 mL) solution of **11** was added the appropriate amine compound (0.44 mmol). The reaction mixture was stirred in the presence of Et_3_N at room temperature. The resultant residue was partitioned in 3 mL water, then treated with 2N HCl to pH 3, CH_2_Cl_2_ was removed under vacuum to precipitate a white solid, that was filtered and the filter cake was washed with water to pH 7, and dried. The crude was purified on a silica gel column with petroleum ether/ ethyl acetate to yield a white powder.

#### 3.4.1. N-[3β-Acetoxyurs-12-en-28-oyl]-3’,4’-difluorobenzylamine *(**12**)*

Compound **11** was reacted with 3’,4’-difluorobenzylamine using the general procedure to give compound **12**. Eluted by petroleum ether/ethyl acetate (v/v) = 8:1; Yield: 50.1%; m.p. 110–112 °C; IR (KBr): 3538, 3002, 1694, 1594, 1497, 1410, 1352, 1035 cm^-1^; ^1^H-NMR (CDCl_3_): δ 6.965–7.108 (m, 3H, Ph-H) 6.194 (d, 1H, NH), 5.251–5.262 (m, 1H, H-12), 4.471–4.504 (br, 1H, NHCH_2_), 4.099–4.132 (m, 1H, H-3), 2.052 (s, 3H, CH_3_CO), 1.089 (s, 3H, CH_3_), 0.925 (s, 6H, CH_3_×2), 0.867 (m, 9H, CH_3_×3), 0.676 (s, 3H, CH_3_); ESI-MS: 624.4 (M+H)^+^; Elemental analysis (%, found) C, 74.71 (74.60); H, 9.00 (9.06); N, 2.34 (2.29).

#### 3.4.2. N-[3β-Acetoxyurs-12-en-28-oyl]-3-morpholin-4-yl-1-propylamine *(**13**)*

Compound **11** was reacted with 3-morpholin-4-yl-propylamine using the general procedure to give compound **13**. Eluted by petroleum ether/ethyl acetate (v/v) = 1:6; Yield: 42.4%; m.p. 98–100 °C; IR (KBr): 3468, 2998, 1694, 1593, 1497, 1421, 1311, 977cm^-1^;^1^H-NMR (CDCl_3_): δ6.369 (d, 1H, NH), 5.286 (s, 1H, H-12), 4.500 (t, 1H, H-3), 3.431 (m, 1H, NCH), 2.051 (s, 3H, CH_3_CO), 1.089 (s, 3H, CH_3_), 0.942 (s, 6H, CH_3_×2), 0.856–0.889 (m, 6H, CH_3_×2), 0.772 (s, 3H, CH_3_); ESI-MS: 626.1(M+H)^+^; Elemental analysis (%, found) C, 74.85 (74.71); H, 10.69 (10.61); N, 4.38 (4.47).

### 3.5. General procedure for the synthesis of N-[3β- butyryloxyloxy-urs-12-ene-28-oyl] amines ***16**,**17***

Compound **14** was obtained from ursolic acid **1** (100 mg, 0.22 mmol) and butyric anhydride by using the same method described for the preparation of compound **2**. A mixture of compound **14** (50 mg, 0.11 mmol) and oxalyl chloride (0.04 mL) in CH_2_Cl_2_ (2 mL) was stirred at room temperature for 20 h. The mixture was concentrated to dryness under reduced pressure. Cyclohexane (3 × 1 mL) was added to the residue, which was then concentrated to dryness to yield crude 3-*O*-butyryloxyl chloride **15**. To a CH_2_Cl_2_ (4 mL) solution of **15** was added an amine compound (0.44 mmol). The reaction mixture was stirred in the presence of Et_3_N at room temperature (TLC control). The resulting residue was partitioned in 3 mL water, then treated with 2N HCl to pH 3, CH_2_Cl_2_ was removed under vacuum to precipitate white solid that was filtered and the filter cake was washed with water to pH 7, and dried. The crude was purified on a silica gel column with petroleum ether/ethyl acetate as eluents to yield a white powder.

#### 3.5.1. Methyl N-[3β-butyryloxyl-urs-12-en-28-oyl]-2-amine *acetate (**16**)*

Compound **14** was reacted with glycine methyl ester hydrochloride using the general procedure to give compound **16**. Eluted by petroleum ether/ethyl acetate (v/v) = 5:1; Yield: 69.5%; m.p. 79–84 °C; IR (KBr): 3388, 2897, 1724, 1664, 1532, 1467, 1311, 1025cm^-1^; ^1^H-NMR (CDCl_3_):δ 6.517 (m, 1H, NH), 5.397 (t-like, 1H, H-12), 4.488 (m, 1H, H-3), 4.075–4.115 (br, 1H, NHCHa), 3.809–3.846 (br, 1H, NHCHb), 3.753 (s, 3H, O CH_3_), 2.273 (s, 3H, CH_3_CO), 1.090 (s, 3H, CH_3_), 0.943 (s, 9H, CH_3_×3), 0.852 (m, 12H, CH_3_×4), 0.701 (s,3H, CH_3_); ESI-MS m/z: 598.6 (M+H)^+^; Elemental analysis (%, found) C, 73.96 (74.08); H, 10.15 (10.25); N, 2.45 (2.33).

#### 3.5.2. N-[3β- butyryloxyl-urs-12-en-28-oyl]-benzyl amine *(**17**)*

Compound **14** was reacted with benzylamine using the general procedure to give compound **17**. Eluted by petroleum ether/ acetone (v/v) = 7:1; 61.8%. m.p. 92–94 °C; IR (KBr): 3498, 2826, 1684, 1612, 1487, 1398, 1108cm^-1^; ^1^H-NMR (CDCl_3_), δ 7.233–7.337 (m, 5H, Ar-H), 6.151 (t, 1H, NH), 5.208 (t, 1H, H-12), 4.552 (d, 1H, Ar-CHa), 4.482 (m, 1H, H-3), 4.155(d, 1H, Ar-CHb), 2.288 (m, 2H, CH_2_CO), 1.078 (s, 3H, CH_3_), 0.945 (m, 9H, CH_3_×3), 0.853 (m, 9H, CH_3_×3), 0.701 (s, 3H, CH_3_); ESI-MS: 616.8 (M+H)^+^; Elemental analysis (%, found) C, 79.73(79.69); H, 10.33(10.28); N, 2.21(2.27).

## 4. Conclusions

We have described the synthesis and basic structure-activity relationship of a series of novel derivatives of ursolic acid as potential lead compounds for the development of new anticancer drugs. Our data suggest that: (1) most UA conjugates with an amino acid methyl ester, amino alcohol, amino alcohols acetate or benzylamine at C-28 and with an acetoxy group at C-3 have greater antiproliferative ability on HeLa cells; (2) compound **13** showed significant anti-tumor activity against HeLa, BGC-823 and SKOV3 cells.
